# How Civil Society Organisations Changed the Regulation of Clinical Trials in India

**DOI:** 10.1080/09505431.2018.1493449

**Published:** 2018-07-13

**Authors:** Salla Sariola, Roger Jeffery, Amar Jesani, Gerard Porter

**Affiliations:** a Ethox Centre, Nuffield Department of Population Health, University of Oxford, Oxford, UK; b Sociology, University of Turku, Turku, Finland; c School of Social and Political Science, University of Edinburgh, Edinburgh, UK; d Independent Researcher and Teacher (Bioethics, Public Health); e School of Law, University of Edinburgh, Edinburgh, UK

**Keywords:** India, regulation, civil society organisations, clinical trials, bioethics, social justice

## Abstract

In 2005 India changed its pharmaceutical and innovation policy that facilitated a dramatic increase in international clinical trials involving study sites in India. This policy shift was surrounded by controversies; civil society organisations (CSOs) criticised the Indian government for promoting the commercialisation of pharmaceutical research and development. Health social movements in India fought for social justice through collective action, and engaged in normative reasoning of the benefits, burdens and equality of research. They lobbied to protect trial participants from structural violence that occurred especially in the first 5–6 years of the new policy. CSOs played a major role in the introduction of new regulations in 2013, which accelerated a decline in the number of global trials carried out in India. This activism applied interpretations of global social justice as key ideas in mobilisation, eventually helping to institutionalise stricter ethical regulation on a national level. Like government and industry, activists believed in randomised controlled trials and comparison as key methods for scientific knowledge production. However, they had significant concerns about the global hierarchies of commercial pharmaceutical research, and their impact on the rights of participants and on benefits for India overall. Pointing to ethical malpractices and lobbying for stricter ethical regulations, they aimed to ensure justice for research participants, and developed effective strategies to increase controls over the business side of clinical research.

## Introduction

As part of the world-wide offshoring or globalisation of clinical trials that dates from the 1990s, there has been a dramatic expansion of global clinical trials and international contract research organisations (CROs) in India. The number of trials with Indian sites grew especially after 2005. That year, following the signing of the World Trade Organization’s (WTO) Agreement on Trade-Related Aspects of Intellectual Property Rights (TRIPS) in 1995, India brought its domestic patent laws into line with WTO requirements. This allowed foreign sponsors to test new drugs in India without fear of copying. In 2005, India revised its pharmaceutical legislation to match the international ICH Good Clinical Practice (GCP) Guidelines – which standardises the conduct of randomised control trials (RCTs) globally and was drawn up by the pharmaceutical industry (Abraham, 2010; Cooper and Waldby, 2014) – to enable global phase II and III clinical trials to take place within the country.

Removing restrictions on global trials was part of a national programme designed to attract trials and encourage Indian pharmaceuticals companies to shift their Research and Development (R&D) from being almost entirely based on ‘reverse-engineering’ of existing molecules to carrying out innovative research on new molecules. Supporters of clinical trials described benefits for India in terms of foreign exchange earnings and Indian involvement in advanced scientific activities. Those promoting India as a trial destination also made bold and, in the event, exaggerated claims about the likely scale of trial activity in India. The market research firm Frost and Sullivan (2012), for example, estimated that Indian clinical trials business was worth USD $485 million (£282 million) in 2010–2011 and would pass the $1 billion (£594 million) mark by 2016.

Local and international promoters within the pharmaceutical sector stressed lower expenses for sponsors because of: low Indian salaries; the existence of a large pool of potential trial participants; and the availability of English-speaking staff. Carrying out trials could also offer sponsoring companies an opportunity to build market access by establishing networks of friendly key physicians. Optimistic estimates of the current and future size of the industry helped it to leverage assistance from the Government of India: direct funding, its support for schemes involving collaboration between industry and academia, sharing state institutions’ infrastructures, and through deregulation itself, for example (Kale and Little, 2007, pp. 605–606).

The changes brought into play a plethora of new actors in the field of clinical research. Kaushik Sunder Rajan has described India’s emergent CROs, international sponsors, clinical hospital sites etc. as a form of capacity building through which India joined the global pharmaceutical industry (Sunder Rajan, 2010). Sariola *et al.* (2015) call this process ‘Big-Pharmaceuticalisation,’ pointing to how the pharmaceutical industry is impacting on how, where and on whom drugs are tested, and changing local industries towards global collaborative and methodological models.

Civil society groups and voices within mass media contested the economic policy leading to the increase of international clinical trials in India from the start. As the number of global trials increased in India (see Table 1), such critiques intensified. In 2013, regulatory reform was demanded by a Parliamentary Committee because of public interest litigation (PIL) by civil society groups and interventions by the Supreme Court of India. Opponents highlighted apparent human rights abuses and damages suffered by trial participants. New rules introduced by the government’s Central Drugs Standard Control Organisation (CDSCO) established new criteria for informed consent from participants, ethics review boards, and the reporting of – and compensation for – cases of injury or death occurring during a clinical trial. Table 1 shows a decline in the number of global clinical trials approved per year because of the public critiques and economic recession in 2010, but in 2013 they dropped considerably. While the numbers have not increased significantly since then, the medium and long-term impacts and possible changes put in place by the election of a new pro-industry government remain to be seen.

**Table 1.** Approvals of trials by CDSCO between 2007 and 2017.

Note: All values taken from the running number of the trials approved for that year.

Source: All sources from the CDSCO website for approved clinical trials, accessed 15 September 2016.

2007–2012: http://www.cdsco.nic.in/writereaddata/DCG(I)%20approved%20clinical%20trial%20registered%20in%20ctri%20website%20(Jan.2013).pdf

2013: http://www.cdsco.nic.in/Forms/list.aspx?lid=1884

2014: http://www.cdsco.nic.in/Forms/list.aspx?lid=1883

2015: http://www.cdsco.nic.in/forms/list.aspx?lid=2093andId=11

2016 and 2017: http://www.cdsco.nic.in/forms/list.aspx?lid=2173&Id=11

Note on the Source: In 2010, the number of approvals by the CDSCO dropped before regulatory restrictions came into force, which was not until 2013. This finding is in line with research using data from clinicaltrials.gov, which also shows a downturn in India around 2010 (Burt *et al*., 2014). Burt et al relate this drop to ‘reports of ethical improprieities, activist protests and departure of international collaborations’ (2014, no pagination). In 2013, with the new regulations, the number drops further, to about a half of the 2011–2012 level.

The national promotion of international RCTs to enhance Indian clinical research thus masked an under-swell of critique from local researchers and activists. Members of civil society organisations (CSOs) expressed critical views regarding the growth of clinical trials in India, but their criticisms were tempered by realistic expectations of what they believed was achievable. Many of the individuals we interviewed were themselves conducting public health RCTs, social interventions or, in the case of one, CRO research. They did not question medical research methodologies, and many believed that in research, an RCT is the gold standard for advancing medical knowledge. Despite this, members of Indian CSOs were critical of how clinical trials had come to India and were being conducted.

This paper seeks to understand these critiques and to link them to the regulatory changes by answering the following questions. How did academic medical researchers, public health researchers and health activists express their opposition to how clinical trials were being conducted? Why and how did they intervene and lobby for tighter regulation? How far did their concerns help frame the PIL that led to increasing stringency in the regulation of trials in India?

Our data show that resistance by academic medical researchers, public health researchers, and health activists in India was primarily against the global hierarchies of the pharmaceutical industry that involves the commercialisation of research, and the associated industry version of capacity building. Their concerns addressed questions of ethics and how to provide social justice for research participants and patients as well for India as a nation. Their efforts to promote human rights protection for research subjects contributed to a drop in the numbers of international trials coming to India. The case highlights how health social movements could resist international clinical trials and mobilise in ways that resulted in increased ethical oversight by governmental regulatory structures (see also Heitmeyer, 2016) and demonstrates that a critical mass can influence the working conditions of the pharmaceutical industry.

## Analytical Perspectives

Voices of Indian activists and public health scholars need to be understood against the backdrop of the emergence of RCTs as the gold standard of clinical research globally, and its international critiques (Cooper, 2011; Timmermans and Berg, 2010; Wahlberg and McGoey, 2007). Increasingly, this design, with its ‘core set of methodologies – randomisation, blinding, clinical equipoise, and informed consent that underpin the claims to universality that aim to expunge uncertainty in clinical practice’ (Kelly, 2008, p. 102), is used across a wide range of systems of knowledge production, disciplines and sectors, from medicine and agriculture to education and economic development. Supporters of the paradigm argue that judging what works should be based on efficacious interventions and good quality data rather than authority, tradition or politics.

Rather than simply being ‘pure and rational,’ however, RCTs have been criticised for trying to keep at bay the ‘messy stuff’ and side-line other kinds of knowledge (Simpson and Sariola, 2012). The RCT methodology has also evolved in response to pressure from the public. Steven Epstein (1996) describes how in the 1980s, LGBTIQ activists and study populations pushed back against US trial regulations that prevented HIV+ people being included in early anti-retroviral (ARV) studies. Activist groups criticised the US Food and Drug Administration (USFDA) regulations for being too rigid and slow in their operations. By lobbying for changes in the inclusion and exclusion criteria of ARV trials, activists shaped how clinical trials have since been conducted and points to how CSOs can actively influence research regulation.

Since Epstein’s seminal work on health social movements, literature on civil society involvement in health, well-being and medical research has developed. Brown *et al*. (2004, p. 50) have produced a typology of these movements: (1) those seeking equitable access to health care and improved provision of health care services; (2) constituency-based movements addressing health inequalities based on race, ethnicity, gender, class and/or sexuality differences; and (3) embodied health movements that address disease, disability or illness experience by challenging science on aetiology, diagnosis, treatment and prevention. These movements often have a ‘disease-base,’ i.e. they focus on conditions such as HIV (e.g. Epstein, 1996; Schneider, 2002), breast cancer (Klawiter, 1999; 2002) or women’s reproductive health (Murphy, 2012). Often, experience of the illness or lack of recognition of a condition is turned into an identity-based struggle (Brown *et al*., 2004). Rabeharisoa *et al.* (2014) show how some patient organisations actively join the ‘quest for cure’ as co-producers of knowledge, shifting the meaning of expertise. Health social movements have mobilised around issues such as access to medicines in the global south (Treatment Action Campaign in South Africa: Schneider, 2002), extended definitions of risk and exposure (breast cancer, see Klawiter, 2002; tobacco, see Nathanson, 1999), and validation from within scientific communities as in the case of complementary and alternative medicines (Hess, 2015).

Mobilising around clinical trials in the Indian context, however, does not fit easily into any one of these categories. In India, many health social movements organised their activities within the framework of ‘rights-based’ approaches, within conceptions of bioethics and social justice, and have focused on health inequalities (especially, but not only, those based on gender). The Jan Swasthya Abhiyan (JSA), an umbrella organisation of CSOs concerned with health advocacy in India, sees its role in terms of drawing attention to the effects of ‘iniquitous globalization on the health of Indian people.’ It ‘locates the campaign to confront commercialization of health care and to achieve “Health For All” by establishing the Right to Health and Health Care.’^1^ Such ideas assume particular salience because, since the liberalisation of the Indian economy, its public health sector has undergone radical changes. Rolling back state services has prompted a vigorous network of advocacy and activist groups – broadly understood as civil society – to campaign for more, and more equitable, public services (Goswami and Tandon, 2013, p. 645). Here we are concerned with civil society activism which includes: ‘Campaigns and policy advocacy efforts [that] focus on the rights of the excluded [and] target negative consequences of rapid economic growth and rise of the free market’ (Goswami and Tandon, 2013, p. 657).

Negative consequences of liberalisation are central to the critiques of clinical trials in India by social scientists, activists, and ethicists alike. In academic discussions, Sunder Rajan (2005; 2010; 2017), Cooper and Waldby (2014), and Prasad (2009) have argued that the quest for profit has led to structural violence for Indian research participants and researchers whereby commercial interests trump ethical interests (see also Fisher, 2013; Jeffery, 2018). More specifically, Prasad (2009) and Sunder Rajan (2017) argue that in the neo-liberal mode, the Indian government has created conditions where it is possible to capitalise on sickness. White (2011) states that instead of focusing on consent as the marker of the ethics of a particular trial, what should be of ethical concern is why, how, and for which populations a drug, vaccine, etc. is made available. Narrowly focusing on bioethical processes in the conduct of a trial – e.g. taking informed consent – legitimises exploitative power, rather than ensures autonomous decision making (White, 2011; Fisher, 2013). Kamat (2014) elaborates these debates further by arguing that clinical research in India should be carried out on diseases that are prevalent in India, so that the drugs that are tested are relevant to the populations where the tests take place, rather than helpful for patients elsewhere.

Issues raised by the emergence of global trials in India identified by public health and bioethics activists and academics are best understood through reflection on the concept of social justice. Although an expansive and broadly defined term without an agreed definition, social justice is emerging in STS literature as an object of analysis alongside bioethics. Reardon (2013) argues that the concept of bioethics has gained more prominent attention through e.g. programmes on the Ethics, Legal and Social Implications of science (ELSI) and the central role that bioethics plays in regulation of science collaborations. While bioethics is defined in narrow terms associated with bureaucracy, audit cultures and legal accountability (Reardon, 2013, p. 180), social justice is seen to address *collective power* (Benjamin, 2016). Reardon (2013, p. 179) proposes that: ‘calls for social justice offer a space for thinking about others. They orient around the collective – around what can come together, and what cannot and why.’

Although Reardon characterises bioethics as an institutional audit-type approach that contrasts with social justice, other definitions can be found. For example, rather than seeing a dichotomy between bioethics and social justice, others have framed bioethics as incorporating ‘justice’ as one of its four foundational principles: autonomy, justice, beneficence, and non-maleficence (Beauchamp and Childress, 1989; Sen, 2009). In particular, justice can be used to scrutinise the distribution of benefits and burdens in research involving human subjects as well as ensuring that people are treated equally (Belmont Report, 1979).

Furthermore, bioethics itself can be conceptualised as a structured form of normative reflection and discussion on ‘what is the right thing to do,’ rather than simply a set of bureaucratic practices. Amartya Sen’s theory of social justice (2009) has deliberation as a central concept. He develops John Rawls’s notion of justice as fairness but insists that justice is not an abstract ideal but is achieved through practical reasoning *that should be directed towards action* reducing injustice and advancing justice (2009, p. ix).

Sen’s theory is helpful for understanding the methods and demands of social movements in India. Ruger (2004) argues that global public health institutions and organisations can contribute to reasoned debates on how to implement general principles (such as the ‘right to health’) and support greater empowerment in the health sector, built on more regulation overall, and the reform of state and social institutions. By this definition, instead of being an individual quality, health justice is realised in social and political arrangements (Venkatapuram, 2011). The case study discussed below analyses the activities of health social movements in India as fighting for social justice through collective action, and engaging in normative reasoning of the benefits, burdens and equality of research.

## Methodology

This paper arises from a project entitled ‘Biomedical and Health Experimentation in South Asia,’ that mapped experimental clinical and public health research in South Asia. The study had ethical clearance from the Ethics Committee of Anusandhan Trust, Mumbai, India; Colombo Medical Faculty, Sri Lanka; Nepal Health Research Council; and the School of Social and Political Science, University of Edinburgh. We investigated the experimental activity taking place, and people’s views about emerging social forms as well as their counter-critiques, in India, Sri Lanka and Nepal. We conducted 337 interviews, 148 of which were in India, 73 in Nepal, 80 in Sri Lanka, and the rest in the US and UK. This paper refers only to pharmaceutical trials in India, which constitute the vast majority of South Asian clinical trials. We interviewed principal and co-investigators, staff of CROs, sponsors, regulators, activists, ethics committee members, and key informants drawn from across the spectrum of those researching or commenting on clinical trial activities.

For this paper, we analyse the views of 25 academic public health and medical researchers and health activists. Those who could be classified as both researchers/doctors and activists were part of nine different non-governmental organisations based across India. Interviewees were prominent in medical research institutions as well as in organisations that have been active in health advocacy such as Low Cost Standard Therapeutics (LOCOST), Sama Resource Group for Women and Children (SAMA), the Forum for Medical Ethics Society, members of the Medico Friend Circle, and Jan Swasthya Abbhiyan. While not all the interviewees would have thought of themselves as being part of a ‘social movement,’ they contributed to the discussions about the arrival of global trials. This suggests that the critiques of international clinical trials went beyond activist networks.

The interviews were conducted in 2011 as the public discussion about clinical trials intensified. We were part of the networks and our early reflections on the project findings fed into the public debates at several conferences and national hearings. Since the formal interviews were conducted, we have maintained informal contact with key activists, and have engaged in close readings of relevant policy documents and publications.

We obtained written informed consent from participants. Interviewees were provided with information sheets regarding the study and its aims. Interviews were in English, recorded, transcribed, anonymised, and coded using Atlas.ti. Transcripts were coded with regular checks for consistency. Codes relevant to this paper include: ‘ethics,’ ‘knowledge production,’ ‘innovation,’ ‘new social forms,’ ‘regulation and governance’ and ‘collaboration.’ The ‘ethics’ coding included any discussions of cultural differences over ethical codes, malpractices or scandals mentioned, post-trial access to drugs as an ethical issue, consent procedures and their value, and altruism as a way of engaging patients or use of doctor–patient relationship to get consent or recruit patients. We also included discussions of ethics review committees: perceptions of their competence, the burden of work, the political pressures they work under, any conflicts of interest and how they were resolved. Any mentions of injury and compensation for trial participants, adverse event reporting as an ethical issue, insurance for trial participants and for trial staff, ‘guinea pig’ as a concept and the ethics of standards of care or ancillary care for trial participants were also identified.

Issues of ‘fairness’ were raised in many of the quotations retrieved following this approach, especially over post-trial access to drugs, and compensation for adverse events. Dominant themes within each category were identified by close reading and re-reading; quotations have been cited where they best exemplify the emergent perspectives. Some quotations have been edited for clarity.

The following sections are organised under key themes that emerged from the review of the interviews: capacity building; commercialisation; and subaltern voices and regulatory changes. For context, we provide a table compiled from the website of the Indian government drug regulatory authority, CDSCO, of trial approvals per year, 2007–2017 (Table 1).

## Clinical Trials and Capacity Development

The Indian government and the pharmaceutical industry claimed that clinical trials deregulation in 2005 would increase collaboration with the international pharmaceutical sector and bring in skills and foreign investment. It was believed that being part of a new research culture would provide its participants with cultural and symbolic capital, which could be transformed into other forms of capital, social and economic, such as networks, salaries, and international positions. Indian activists and researchers recognised that local motivations to collaborate are important but pointed out that ‘capacity to do what?’, remained a loaded question. Several of our interviewees saw that those who were working in institutions drawn into collaborations with Big Pharma, CROs, study site hospitals, etc. – at times their colleagues – were providing relatively unskilled labour to produce global data rather than becoming innovators. When asked about what kind of capacity was being built by the new research culture, a dean of medicine and a public health researcher answered as follows:

It builds capacity in terms of making people understand the process – consenting, randomisation, recruiting subjects, strategies around that – and that is some capacity building. The problem with industry driven studies is that the protocol is written by someone else, and you’re just an implementing agency. You don’t understand the nitty-gritty of writing a protocol, doing research, [and] asking the questions yourself. (Academic public health researcher 2011)

Other interviewees were also concerned that skills drawn together around clinical trials replicated global hierarchies, and interviewees – explicitly or implicitly— highlighted social injustices. They were critical of the direction in which the industry was pushing clinical research and were attentive to power relations in such collaborations. They saw Southern partners being left with handmaiden roles as facilitators rather than as knowledge leaders. Another eminent researcher attached to a university department in public health described clinical trials research as follows:

Academics have started to shape themselves based on clinical trials, which is really pre-cooked research, it is not research, it is operations, and I think it is a dreadful thing that has happened to India. India is becoming a service centre-economy for the middle class, extending to academics and research. (Academic public health researcher 2011)

This interviewee criticised what could be likened to the ‘coolie’ (a pan-Indian term for precarious day labourers) role in clinical research and how Indian academics were reduced to servicing a research industry not driven by scientific questions set locally but conforming to pre-defined research questions set in – and benefiting – the global North.

A retired senior researcher in public health drew an analogy between Indian trials research culture and the outsourcing of office cleaning, suggesting that staff in janitorial companies can never progress beyond managing other janitors according to externally generated rules:

There is a striking resemblance in what clinical trials are doing and what these guys are doing. We are building a bunch of servants with different capabilities but none of them will ever come into the leadership role. (Academic public health researcher 2011)

Another researcher and sponsor, part of a national public health research organisation that conducted cluster randomised public health interventions, elaborated on this idea, suggesting that Indian researchers *could* break the glass ceiling in international collaboration but, based on his/her long-term experience, this took much longer than was reasonable:

At the beginning, impact evaluations were all northern academic driven. Southern participants were really just data collectors. Then, after doing some 300 projects the young southern researchers have also matured and are sort of taking on leadership. But it doesn’t need to take 10 years for that to happen. (Academic public health researcher 2011)

The move towards clinical trials was seen to be developing commercial rather than scientific research capacity. Interviewees’ views resembled those of Melinda Cooper (2011), for instance, who has argued that a clinical trial is essentially not ‘research,’ but is more aptly described as a test, the result of which simply validated a prior set of findings or assumptions. Interviewees described power differences in the emerging research networks where existing capacities were oriented to carrying out peripheral tasks in the CRO sector. They could be members in a global science network but could not move towards being more central to knowledge production. The high-powered roles of Indian biotech staff returning from US or UK to run these networks were not mentioned as evidence to the contrary. In the next section, we elaborate further on social justice-related concerns regarding the commercialisation of research cultures.

## Commercialisation of Research

Leading members of Indian CSOs did not see industry-driven R&D and increasing commercialisation favourably. Commercialisation, they said, had a corrupting effect. An academic researcher actively involved in public–private partnerships with Big Pharma using RCTs described the institutional challenges of trying to collaborate in a field where certain sponsors can pay significantly higher fees to their investigators:

In the early days, many institutions were getting to know drug trials, particularly company- sponsored drug trials. They realised that they are a good source of income. So that was a big challenge that affected us, being an academic centre. With more than half of our studies with minimal or even very tight funding, it became a challenge for us to match the expectations of those kinds of funding. How did different institutions respond to this? Two ways: one, ‘here’s an opportunity for us to do good quality collaborative research!’ But the moment people began to see money in this, then, two, it turned a little bit the wrong way, wherein money and power came into the forefront, and research and science took the backseat. (Academic researcher involved in commercial research 2011)

Academic researchers saw that increasing amounts of money in the hands of researchers lured good PIs away from academic research. It was difficult to compete with promises made by commercial sponsors. Non-applied academic research questions could become side-lined because they lacked monetary potential. Academic freedom and basic ‘science for science’s sake’ were at stake. Scientists had concerns about academic freedom and non-commercially viable research, in the face of the lure of financial gain, reflecting age-old concerns between academic research and industry.

To guarantee national interests in this climate was not straightforward. Here the responses reflect the complexities of the changing pharmaceutical landscape. One interviewee described the changes in research culture from the perspective of the health movement that s/he was part of. Since 1978 they had maintained public discussions regarding the global architecture of health politics and economics, including e.g. intellectual property rights, generic pharmaceuticals and the privatisation of science and technology infrastructures. They aimed at de-mystifying science and technology to enable people’s participation in discussions about health policy-making:

Looking at India, the issue of clinical trials brings forth both questions of the potential of science and what it can do for you, but also more importantly especially in the Indian context the way scientific research today requires much larger societal oversight and control in a situation where it’s becoming more and more complex. So in that sense it’s becoming more and more difficult for the public to grasp what’s happening and be able to make sense of how they need to react to this. (Doctor and science activist 2011)

Biomedical research has the potential for furthering public interests, then, but commercialisation brings in complexities that were difficult to grasp and govern. For instance, commercial researchers could not guarantee that new knowledge and research benefits would stay in the country when such research was aimed at global markets. Activists were largely correct in this regard. In a study of 224 trials with an Indian site listed on clinicaltrials.gov in 2010, 133 did not result in marketing approval in India or the EU/USA (i.e. they failed). Of the 91 that succeeded, 55 drugs were approved for sale in India, leaving 36 drugs approved in the EU or USA but not in India. This happened despite the Indian drug regulatory authority’s requirement that sponsors guarantee that they will market the drug in India after conducting a trial involving Indian participants (Limaye *et al.*, 2015). Moreover, many drugs that are made available in India remain too expensive for the poor (Nadimpally *et al.*, 2016).

These discrepancies have led Medico Friend Circle, to which many of our respondents belong, to argue in their mission statement that:

We believe that medical and health care must be available to everyone irrespective of her/his ability to pay  …  Also that medical intervention and health care be strictly guided by the needs of our people and not by commercial interests. We, therefore, work towards health care services based upon human values, concern for human needs, equality and democratic functioning.^2^

The statement highlights their concern about the commercialisation of health and their embracing of the idea that the cost of a drug should not determine access to medicines: this should be a matter of human rights. A further example of resistance to the revenue-based pharmaceutical field is LOCOST, a non-profit charitable trust established in 1983 to provide drugs in pro-poor ways. Iyer (2012) explains that LOCOST’s goal was to demonstrate that quality drugs can be provided at affordable prices, by making generic formulations and distributing them via local social service organisations. One of the founder trustees stated their principles – to ensure equitable access to medicines*:*

We don’t sell drugs in the main market. We started this organisation because a lot of friends working in remote rural areas work with very poor people. They didn’t have access to good quality medicines at reasonable prices, so we started with that. There was a lot of demand. (Drug developer and activist 2011)

LOCOST does not conduct clinical trials. In a conversation regarding research ethics, the interviewee illustrated the argument of this paper poignantly by stating that RCTs are important but raise ethical problems.

Obviously, we need clinical trials. I really don’t have any alternatives in mind in terms of advancement of knowledge, validation of knowledge and placebo control, double-blind clinical trials or comparative trials. It raises a lot of problems but even with all these problems it is still the best way there is to validate the statement. So you can’t really throw the baby out with the bathwater. But there are ethical issues, we need to think of the ethical minefields in these processes. Pharmaceutical companies and especially contract research organisations in India are notorious for lacking ethical oversight if nobody is looking. (Drug developer and activist, 2011)

Rather than denying the value of RCTs, the interviewee reflected on the ethical problems and then suggested further points of regulation, some of which were included in the 2013 legislation.

So we need a lot of regulatory oversight on ethical issues where clinical trials are conducted, by which I mean four principles. First is accountability, if anything goes wrong or goes right; second is transparency as far as possible. I agree that there are some things called trade secrets, but I am not asking about trade secrets. If something goes wrong, I should be able to have enough data to place responsibility. The third thing is liability, there should be a law of liability stating who all should be accountable; that should be very clear. And fourth is: I feel that our people in India, and especially poor people and tribals, who are often the clinical targets in India, are easy targets. They need to be given special care by the government and we need to have systems in place so that they are not exploited. If anything goes wrong, they need to get compensation. We don’t have that kind of regulatory system. We don’t have the resources to do research perfectly. As far as ethics is concerned we are not very evolved. (Drug developer and activist, 2011)

On the one hand, LOCOST directly questions the underlying structures of private pharmaceutical companies, such as patents, intellectual property, and high prices of drugs that result from expensive research and hyperbolic claims of their efficacy. On the other hand, the interviewee criticises the Indian regulatory framework in place prior to 2013, where the vulnerability of certain populations was exposed. S/he proposed amended regulation to prevent poor and disenfranchised people being taken advantage of. There was no questioning of the system of knowledge production through the RCT as such. On the contrary, the interviewee suggested that – despite the commercialised use of RCTs by Big Pharma – structured comparison remains the best method to test if something works, but that in India, more stringent regulation was needed.

Another public health researcher, who conducts evaluation and implementation studies and runs an NGO supporting health care access and rural health development, also emphasised the interests of the public sector and people’s access to health care. In a resolute statement, s/he illustrated a common feeling that CSOs needed to do something about the regulatory situation:

I think we have to be in the real world, so I don’t think we can take a very ideological and deterministic position, which we do and which I myself have taken in the past. My feeling is that it is very important that the public interest has to be strengthened. Even though I have strong reservations for all these public-private partnerships, my overall feeling is that we must increase the stewardship and regulation of the government and not just see it as regulation but to try and promote the public interest which is what the government has been voted to do. So in that sense, I’m a little bit less deterministic than I used to be. If you had asked me ten years ago, I might have said all research should be done by the Indian Council of Medical Research, but I realise that the way the world is going today you are going to have industry, you are going to have R&D, and one has to regulate it. I don’t think just the legal approach helps. We also need to strengthen professional leadership and equity, patients’ rights, patient’s charters and things like that. So again, I think it is a question of being in dialogue and ensuring that the public interest, safety, and equity, are raised, rather than only talking about the source of funding. (Public health researcher and activist 2011)

The private sector has shown greater commitment to generic drugs than the public sector, s/he said, to ensure that poorer Indian and global populations could access drugs and health care, such as the cheap ARV therapies marketed by CIPLA, an Indian commercial, generics pharmaceutical company. CSOs lobbying for change were struggling with these contradictions and the interviewee proposed that simple ‘good/bad’ dichotomies were no longer useful or accurate. In this context, drawing conclusions based on the origin of funding (private-bad vs. public-good, or Indian-good vs. foreign-bad) no longer worked.

Like other interviewees, s/he said that local sacrifices should be clearly outweighed by local advantages. Balancing local interests was difficult as Indian ownership was no longer sufficient to ensure that benefits were provided locally. Nor was it always clear how international collaborations, foreign sponsors and private efforts undermined national benefit, or who could be trusted to direct research in morally acceptable ways. For these interviewees, it was no longer clear what national benefit meant, nor how it could be guaranteed.

All this called for dialogue between civil society and the state, and consideration of the ethics of the new situation. Despite the overall resistance to the commercial pharmaceutical sector, the members of CSOs recognised that these trials had come to stay, and that strategies were needed to improve regulation. In sum, the question of the terms and conditions within which clinical trials should be carried out did not lead to a call for refashioning of RCTs or, more broadly, evidence-based medicine. Rather, the new, commercialised situation required new ethical considerations and regulatory frameworks. In less than ideal circumstances, members of civil societies put their dislike of global trials to one side in order to find a compromise that involved tighter regulation of the emerging research to ensure rights of research participants.

## Subaltern Voices and Regulatory Changes

CSOs engaged with research ethics in India highlighted lacunae in the regulation and practice of clinical trials in place after the deregulation of trials in 2005.^3^ We finish this paper with a meta-critique presented by one of the researchers who linked global clinical trials with post-colonial relations in technoscience, which elsewhere has been described as neo-colonialist (Nundy and Gulhati, 2005). His/her reflection poignantly describes how global tensions with the pharmaceutical industry impact on participants and advocacy groups. Before the new regulations were in place, s/he described anger among CSOs:

There is the basic reality in communities that people have been perceived to have been coerced (into research) and they did die (as a result). This is such an insistent reality that I understand why outrage alone seems to take centre-stage and why the outcome feels like a rejection of trials. Subaltern voices are not heard, by and large, that’s what we mean by them being subaltern voices, and so that when they are heard they will be sweepingly stated. When you’re not heard, on the rare occasion on which you manage to be heard, you will not sound nuanced and comprehensively aware of the many aspects of the situation. Still less will you be enthusiastic (about) establishing a reasonable dialogue because, really, you’re using that one rare opportunity simply to be heard in protest. I cannot bring myself to say to people who are protesting about clinical trials, that they shouldn’t be doing this, I cannot: it’s almost an emergent property of the situation. So, yes, a strident rejection (of international RCTs) doesn’t do the problem a great deal of practical solution but it is well-merited and understandable, so I tend to want to say that they might use the strength of the outrage to make structural changes. It’s an old left strategy that doesn’t necessarily work but since I don’t have anything better  …  . (Biomedical researcher and activist 2011)

The interviewee portrays the subaltern voices – the multiple voices of public health researchers and health activists in India – shouting to be heard in outrage. The anger was palpable in the public meetings organised by the activists that we attended. But in more measured terms, CSOs’ strategic arguments have helped bring about change. A persistent critique has been that the planning and justification by the Indian government for the deregulation of clinical trials made little mention of benefits to patients or populations; rather, foreign investment and collaboration were foregrounded (Bajpai, 2013). Various arguments have been raised around particular studies such as the demonstration project (or a Phase IV trial – accounts differ) involving vaccination against Human Papilloma Virus, starting in 2007, amongst 9–15-year-old girls in Andhra Pradesh and Gujarat, many of whom were living in hostels and whose wardens gave mass consent (Parliament of India, 2013). Another set of problematic trials took place between 2004 and 2008, when patients at the Bhopal Hospital for victims of the gas leak disaster of 1984 were involved in trials, often without their knowledge (Lakhani, 2011). A 2012 Parliamentary Report initiated by civil society groups brought the ethical concerns to wider public notice and set in train reforms to the procedures for the approval of clinical trials nationally (Parliament of India, 2012).

Many activists and researchers we interviewed felt that lax regulations prior to 2013 were an invitation for international pharmaceutical companies to establish their operations in India and that the government office that approves these studies was ill-equipped to regulate them. The relaxed regulatory environment and lack of emphasis on the rights of participants led a medical researcher and social worker to elaborate on ethical variability (see also Petryna, 2009):

Now, I think one ethical issue in this is that when you have international collaboration, how many international collaborators are collaborating with India because the ethical guidelines and controls are much less? So in a way, in my general feeling, though it might be a sweeping statement, a lot of international collaborations are finding surrogate partners in developing countries and are often not aware of the local levels of ethical regulation. (Medical researcher and social worker 2011)

The reduction in the number of international trials in India post-2013 indicates that this flexibility had indeed encouraged companies to outsource their activities to India. A common concern of activists was that private ethics committees acted as rubber-stamps for international (or local) pharmaceutical companies (see also Simpson *et al.*, 2015). Our interviewees accused ethics committees assigned the role of reviewing clinical trials of having become money-minded, when their role should be to protect vulnerable populations and defend the interests of the Indian nation.

In the climate of lax ethical regulation of trials, one of the NGOs involved, SAMA, describes its reasons for being involved in the campaign for the rights of clinical trial participants as follows:

Biomedical research such as clinical trials are filled with many complex ethical issues, which includes quality of health care, post-trial access to medicines for research participants as well as general population, improper compensation mechanism in any incident of trial related injury etc. With the growing number of clinical trials in India, there have been many examples of clinical trials that have taken place with disregard to ethical aspects and participants’ rights. There exists a striking lack of transparency at the levels of planning, design and implementation of these trials. In the absence of adequate regulatory jurisdiction and systematic review of the industry, the reliability and validity of drug research in the country is jeopardized. SAMA is considering health a fundamental human right, and advocates for a rights-based approach to health care for trial participants.^4^

The actions taken by the CSOs to influence government procedures included several PIL petitions, notably by NGOs Swasthya Adhikar Manch (SAM) over irregularities in Bhopal, and by SAMA, Drug Action Forum, and Delhi Science Forum over the HPV vaccine. Derived in part from US experience, in India PIL petitions have been used since the 1970s to institute actions around environmental, social and health issues, with differing effects (for more details, see Divan, 2016; Terwindt, 2014). The judicial interventions that CSOs could induce led to various ways of tightening the regulation of clinical trials registered with the Government of India’s trial registry.^5^

In February 2013, under an amendment to Schedule Y of the Drugs and Cosmetics Act, 1945, the Ministry of Health and Family Welfare required all ethics committees to be registered with the Drugs Controller General India (DCGI) before reviewing and approving a clinical trial. This order set out conditions for ethical review, the composition of ethics committees, and mechanisms to prevent conflicts of interest. Other rules dealt with documentation and communications, confidentiality and the maintenance of records to allow for investigations following adverse events. The possibility for trials to ‘shop around’ for more pliable ethics committees has been at least partially addressed through the centralised registration of ERCs. In registering a trial, sponsors must report all applications for ethical review (Central Drug Standard Control Organization, 2013).

Major concerns among the interviewees included ‘informed consent,’ and its implications for access to health care. A doctor and science activist weighed the importance of trials to medical knowledge production with access to health as follows:

For me the major ethical problem in having so many clinical trials in India  …  I’m not against having clinical trials in India. As a science activist I won’t argue that you don’t need clinical trials … But for me the biggest problem of clinical trials in India is related to the public health system. The fact is that a majority of people are in a situation where clinical trials offer them the only way in which they can access treatment. I think that goes against the notion of informed consent. But for me that is entirely meaningless because even then there is a major element of coercion because of the systemic reasons to do with the fact that the person is denied health care by the public system, which forces him to enrol. (Doctor and science activist 2011)

Civil society groups lobbied for the inclusion of audio-visual recording of the informed consent process, and a draft rule was published that would make this compulsory for all clinical trial participants. This rule was later watered down by limiting the audio-video requirement to the enrolment of ‘vulnerable’ patients/participants in clinical trials using New Chemical Entities or New Molecular Entities. What must be said to trial participants has been more clearly specified, including (if it is a placebo-controlled trial) that the placebo will not have any therapeutic value.

The most contentious aspect to the reforms has been the payment of compensation for clinical trial-related injuries or death. The reforms aimed to clarify the procedures for assessing clinical trial related injuries and deaths, and to ensure that appropriate compensation is paid to participants or their heirs and beneficiaries. However, uncertainties remain about the scope and extent of legal liability and how new rules might be implemented (Srinivasan, 2015).

Heightened standards of rights protection for trial subjects, such as higher levels of compensation for injury and post-trial access to medicines, can increase the costs of running a trial. Compensation in case of injury was stipulated in the new legislation, while obligatory access to medicines was not. There is evidence to suggest that the reason why obligatory post-trial access was dropped from the new legislation had to do with the practical implications that access to medicines would have on increasing costs to sponsors and CROs (Porter, 2017). Such costs were feared to make India too expensive and thus a less attractive site for clinical trials. Post-trial access remains a recommendation in the guidelines but is at the discretion of sponsors and CROs. Activists have nonetheless continued to lobby for the importance of post-trial access, especially to enhance the well-being of poor populations in India (Nadimpally *et al.*, 2016).

As a result of the regulatory changes, the number of clinical trials approved by CDSCO dropped by a third in 2011–2012 from the figure for 2010 (see Table 1). As Burt *et al.* (2014) also argue, the main contributory factor was the spotlight that activists and the media directed towards clinical trials in India. With the new legislation in 2013, approvals plummeted to a fifth of what they were at their peak in 2010. Several international companies, including the American CRO Quintiles and AstraZeneca, closed units in Hyderabad and Bangalore. Faced with these threats to the trials industry, the Drugs Controller General of India suggested some easing of the proposals and also announced that a shortening of the time-frame for approval of new clinical trials to six months from their submission.^6^

In sum, civil society groups were key to raising ethical concerns regarding global RCTs and bringing attention to how clinical trials were conducted in India. The public discussion about experimentation and novel modes of regulation followed a vibrant social debate and a government-civil society dialogue. CSOs successfully highlighted ethical issues and questions of social justice, protection of participants and opposition to exploitation that went beyond the previous tick-box approach to ethical assessment. They highlighted controversial trials to leverage public attention and politicised them by bringing them to the attention of the Indian Parliament. Using tools such as blogs, publications and the PIL, CSOs’ members insisted on discussions of social and moral values. Their use of ethics and patient rights was a rhetorical tactic to counter industry arguments and pushed the government to take action to regulate better the clinical trials taking place in the country. They maintained strategic attention on public interests and prevention of exploitation while ensuring that lines of communication stayed open. Rather than a demand to ban all clinical trials, as a couple of activists in private told us they would prefer, it is likely that this active engagement worked much better as a strategy to change regulation.

Theirs was not a call for scientific pluralism, however, or a wholesale denial of clinical research methodologies. Researchers and activists supported controlled experimentation as a methodology and saw comparison as a scientific gold standard. Perhaps because of their non-fundamentalist stance and the language of medical research that they shared with their interlocutors in the industry and in government, they were able to develop a coherent strategy to bring clinical trials under more regulation. Thus they were able to craft an alternative direction to the clinical trials industry by global and local pharmaceutical companies in India.

## Conclusion

In this paper we have described the ethical deliberation and action taken by CSOs in India in response to the increase of international clinical trials after 2005. Unlike other academic observers of international clinical trials in India, we have focused not on the exploitative dimensions of the increase, but rather on CSOs’ activities challenging the views of the clinical trials industry and government regulators. CSOs played a major role in changing research regulations in 2013, in turn leading to a decline in clinical trials in the country.^7^

Their success marks a noteworthy example of action against neo-liberal pharmaceutical regimes. CSOs raised issues about access to medicines, problems in ethical regulation, and the overall commercialisation of research within unequal collaborative structures. The new regulations directly addressed CSOs’ concerns regarding ethical oversight and slowed down (at least temporarily) the trend towards commercialising research.

The example stands out among health social movements in two distinct ways. Unlike much of the past literature on health social movements, the activism against global trials in India did not come from identity or illness-based movements of patient activists (Rabeharisoa *et al.*, 2014). Nor were our interviewees lay people engaged by Global Health sponsors or policy-makers (Reynolds and Sariola, 2018). Rather the Indian CSOs were a diverse group of medical professionals who often ran humanitarian efforts alongside their academic or clinical practices. Although they were critical of the social justice implications of trials, they shared central notions regarding the RCT methodology with the industry conducting trials and those who were governing them, i.e. the government body in charge of national research regulations.

CSO action criticised how the global pharmaceutical industry was pushing the field of biomedical research towards commercial testing, thus undermining a socialist pharmaceutical sector with a vibrant generics industry that previously had made cheap drugs available for the masses (Sunder Rajan, 2010; Sariola *et al.*, 2015). This shift towards commercialisation, they thought, created and recreated global injustices for research participants and researchers alike. Especially for those with a strong Marxist, humanitarian, or social work ethos, commercialisation was seen to be taking the field of medical research in the wrong direction.

While Indian CSOs did not want to ‘Stop the march of knowledge,’ as summarised by one of our interviewees in 2011, they did redirect clinical trial activity in the country. CSOs in India were successful in shaping research regulation, partly because they shared the language and principles of research methodology with government innovation policy-makers and the clinical trials industry. Unlike resistance to RCTs as hegemonic forms of knowledge production, as expressed in the STS literature and other articles in this special issue, Indian activists had confidence in RCTs as a method. Industry, government and our interviewees saw comparative methods as central to defining what works; they valued what medical research can deliver.

Despite the methodological overlaps, however, the CSOs emphasised wider ethical concerns that specifically affect low-income populations, facing an Indian government that has been strongly pro-industry since 2014. When CSOs used various strategies such as PIL to put pressure on the government to change research regulation, they sought to enact social justice through public reasoning and action, as in the strategy proposed by Amartya Sen (2009). Embodying this pragmatic account of justice through their campaigning, CSOs lobbied the government to change ethical guidelines concerning patient and participant rights, consent, and compensation following injury or death. Indian CSOs were able to push for regulatory changes that addressed rights of trial participants on their own terms. With an Indian government since 2014 that is strongly pro-industry and investment, however, the battle for more equal relations in the industry is not over. On-going ethical debate is needed to address the question raised in 2011 by an ethicist-activists who was concerned about national benefit: ‘What’s in it for us?’

## Notes

1. http://phmindia.org/about-us/ (accessed 15 September 2017).

2. http://www.mfcindia.org/main/perspective.html (accessed 15 September 2017).

3. Activists in India have commented on ethics of clinical trials before the 2005 deregulation of international pharmaceutical companies: an early instance is the 1993 study in Gadchiroli on alternative modes of providing neo-natal services, in which infants in control groups did not receive best available alternative care (Angell, 2007; Bhutta, 2007). The ethical-technical details that were discussed around this study included the definition of standard of care. How should control groups be chosen in very impoverished contexts where access to such best care is not available but when second best options are being tested (Bandewar and John, 2011; see also Bang, 2010)? As in the ARV trial scandals in Africa, purists argued that standard of care should be the same everywhere irrespective of the sponsor, location and population, while pragmatists argued that somehow local realities need to be improved and that research is needed for that (Haire, 2013; van der Vliet, 2004).

4. http://www.samawomenshealth.in/ethics-in-clinical-trials/ (accessed 15 September 2017).

5. Much of the activity of CROs in India is concerned with bio-availability and bio-equivalence trials, which largely escape such regulation. A window on how much work is being carried out for producers of generic drugs is provided by the accusations of malpractice made against GVK Biosciences, in Hyderabad, which led to the withdrawal of recognition by the European Medicines Agency of some 700 drugs made in India (Sariola, 2015).

6. http://www.sgpgi.ac.in/sop/Action_RR_Choudhury_Committee__06.11.2013.pdf, p. 3 (accessed 14 September 2016).

7. For an analysis of another politicised health social movement in India see the motion developed to free Binayak Sen, a doctor who worked in social medicine in Chhattisgarh, a very impoverished tribal area, and was arrested for supporting a Maoist political movement (Sinha, 2015). There were some overlaps in organisations that mobilised against clinical trials and for freeing Binayak Sen.
